# Measuring trust in artificial intelligence: validation of an established scale and its short form

**DOI:** 10.3389/frai.2025.1582880

**Published:** 2025-05-09

**Authors:** Melanie J. McGrath, Oliver Lack, James Tisch, Andreas Duenser

**Affiliations:** ^1^Commonwealth Scientific and Industrial Research Organisation (CSIRO), Clayton, VIC, Australia; ^2^School of Psychology & Australian Institute for Machine Learning, University of Adelaide, Adelaide, SA, Australia; ^3^School of Psychological Sciences, University of Melbourne, Melbourne, VIC, Australia; ^4^Commonwealth Scientific and Industrial Research Organisation (CSIRO), Sandy Bay, TAS, Australia

**Keywords:** trust, artificial intelligence, automation, human-AI teaming, collaborative intelligence, psychometrics, measurement, validation

## Abstract

An understanding of the nature and function of human trust in artificial intelligence (AI) is fundamental to the safe and effective integration of these technologies into organizational settings. The Trust in Automation Scale is a commonly used self-report measure of trust in automated systems; however, it has not yet been subjected to comprehensive psychometric validation. Across two studies, we tested the capacity of the scale to effectively measure trust across a range of AI applications. Results indicate that the Trust in Automation Scale is a valid and reliable measure of human trust in AI; however, with 12 items, it is often impractical for contexts requiring frequent and minimally disruptive measurements. To address this limitation, we developed and validated a three-item version of the TIAS, the Short Trust in Automation Scale (S-TIAS). In two further studies, we tested the sensitivity of the S-TIAS to manipulations of the trustworthiness of an AI system, as well as the convergent validity of the scale and its capacity to predict intentions to rely on AI-generated recommendations. In both studies, the S-TIAS also demonstrated convergent validity and significantly predicted intentions to rely on the AI system in patterns similar to the TIAS. This suggests that the S-TIAS is a practical and valid alternative for measuring trust in automation and AI for the purposes of identifying antecedent factors of trust and predicting trust outcomes.

## 1 Introduction

Artificial intelligence (AI) is rapidly transforming workplace dynamics, offering unprecedented opportunities to enhance productivity, decision-making, and innovation across industries. However, effectively integrating AI systems into organizational settings presents complex challenges, including ethical considerations and workforce adaptation. With the increasing prevalence of AI in workplaces, there is a pressing need to understand the psychological dimensions of human trust in these systems.

Trust is critical to human willingness to rely on technology, and its importance only increases with the greater complexity and opacity of modern AI applications (Hoff and Bashir, 2015; Lee and See, 2004). However, it is not simply a matter of more trust breeding greater reliance and achieving better results. To work safely and effectively with technology, user trust must be appropriately calibrated to the capabilities of the system (Parasuraman and Riley, 1997). Where trust exceeds the capability of the system, human complacency may lead to misuse, with outcomes ranging from inconvenient to catastrophic (Robinette et al., 2016). Conversely, a lack of trust in a trustworthy system risks disuse, leading to reduced productivity and lost resources. Identification of the optimal level of trust for appropriate calibration is thus crucial to the safe and effective use of automated systems. How to measure trust is, therefore, a fundamental question for the integration of AI into organizational settings.

Self-report scales and items measuring trust in technology tend to proliferate in the literature, with a recent narrative review identifying 30 separate scales for quantifying aspects of the trust construct and a plethora of single-item measures (Kohn et al., 2021). In response, researchers have called for increased psychometric validation of the scales in use, with a particular focus on the generalizability of established technology trust scales to various contexts (Chita-Tegmark et al., 2021; Kohn et al., 2021). Our study responds to these calls by conducting a psychometric validation of the most widely used self-report measure, Jian et al.’s Trust in Automation Scale (TIAS) (Gutzwiller et al., 2019; Jian et al., 2000; Kohn et al., 2021).

## 2 The Trust in Automation Scale (TIAS)

Development of the TIAS (Jian et al., 2000) involved cluster analysis to identify groups of words linked to the concept of trust in machine systems. From that analysis, two clusters were identified, and 12 scale items were extracted (e.g., “The system is deceptive,” “I am confident in the system”; see Table 1 for the full list of items). Jian and colleagues proposed that the two clusters represent the poles of trust and distrust lying along a single dimension.

**Table 1:** Items in the TIAS (Jian et al., 2000).

TIAS
1.The system is deceptive.*
2.The system behaves in an underhanded manner.*
3.I am suspicious of the system’s intent, action, or output.*
4.I am wary of the system.*
5.The system’s action will have a harmful or injurious outcome.*
6.I am confident in the system.
7.The system provides security.
8.The system has integrity.
9.The system is dependable.
10.The system is reliable.
11.I can trust the system.
12.I am familiar with the system.

*Reverse scored items.

Despite its wide use over more than 20 years in relation to a diverse range of automated systems, the TIAS has been subjected to relatively little psychometric validation. Spain et al. (2008) conducted a factor analysis of the TIAS and observed two correlated factors reflecting the trust and distrust clusters proposed by Jian et al. (2000) but conducted no other tests of validity. Perrig et al. (2023) conducted factor analyses that largely supported Spain et al.’s (2008) findings of an oblique two-factor model but evaluated no other forms of psychometric validity.

The first purpose of this study is to conduct a comprehensive psychometric evaluation of the TIAS and test its generalizability to contemporary forms of AI that may feature strongly in public perceptions (e.g., autonomous vehicles) or be regularly encountered (e.g., recommender systems).

### 2.1 The Short Trust in Automation Scale (S-TIAS)

Administering a scale with 12 items interrupts and may influence human–AI interaction, and consequently, the TIAS is typically only used to measure trust at the end of an experimental block or task (Kohn et al., 2021). However, investigation of many important questions regarding the dynamics of human trust in AI, such as the nature of temporal trajectories of trust development (Guo and Yang, 2021), trust loss and repair (Baker et al., 2018), and mechanisms of trust calibration (de Visser et al., 2020), relies on repeated measurements in a range of contexts. Consequently, it is increasingly necessary to identify measures of trust that are time-effective, practical, and rigorous (National Academies of Sciences, Engineering, and Medicine, 2022; Kohn et al., 2021).

In a narrative review of the measurement of trust in automation, Kohn et al. (2021) observe that researchers most frequently used custom trust measures, many of which consisted of only a single item. While recognizing the practicality of such measures, the authors noted the deleterious impacts on external confidence in trust findings and the difficulty in translating those findings between studies. They recommended that in seeking more parsimonious and practical self-report measures of trust, researchers look to extract items from existing scales that have been subject to appropriate psychometric validation (Kohn et al., 2021). The second purpose of this study is to respond to this need by extracting and validating a 3-item version of the TIAS.

## 3 Validation of the Trust in Automation Scale (TIAS)

Trust judgments are largely based on evaluations of the trustworthiness of the target (Mayer et al., 1995). Trustworthiness may be assessed on several dimensions, key among them are ability (performance) and integrity (whether the system operates fairly and in accordance with the goals of the user) (Hoff and Bashir, 2015; Mayer et al., 1995). Our first set of research questions concerns the ability of the TIAS to reflect the differences in trust that are expected when interacting with a system that demonstrates a greater or lesser degree of trustworthiness (Lee and See, 2004).

In Study 1 and Study 2, we also test the convergent and predictive validity of the TIAS. Convergent validity of a measurement instrument speaks to whether statistical relationships are observed between the construct measured by the scale (in this case, trust) and other constructs to which it is theoretically expected to be related (Gregory, 2013). Many models of trust in AI identify a dispositional tendency to trust as predictive of trust in a particular system (Hoff and Bashir, 2015; Lee and See, 2004). However, it is not always specified in these models whether this propensity to trust is specific to other humans as targets or to machines. Consequently, we measured both human trust propensity (HTP) and machine trust propensity (MTP) across the two studies and expected to see them positively related to scores on TIAS.

As indicated earlier, trust is considered important in human-technology interaction due to its role in predicting reliance on AI (Lee and See, 2004). A valid measure of trust in AI should, therefore, result in observations of a positive association between reported trust and intention to use a given system. Across our two studies, we asked participants about their intention to use the AI applications in the vignettes. We anticipated that trust scores would not only predict intentions but operate as a distinct construct predicting unique variance over and above other variables.

### 3.1 Study 1: performance

In Study 1, participants were shown a vignette describing interaction with one of three AI applications: a self-driving car, a virtual assistant providing support in choosing a mobile phone plan, and a diagnostic medical app. The trustworthiness of AI system performance was manipulated between participants. Our first hypothesis concerned the sensitivity of the TIAS to these manipulations of performance. In hypothesis 1a, we anticipated that participants in high-performance conditions would report significantly higher levels of trust in the TIAS than those in the low-performance condition. In hypothesis 1b, this result would be generalizable and observed for all three AI applications.

Hypotheses 2 and 3 are related to the convergent validity of the scale. Hypothesis 2 was that there would be a significant positive association between trust scores measured by the TIAS and propensity to trust humans (HTP), while Hypothesis 3 was that we would similarly observe a significant positive relationship between trust scores measured by the TIAS and reported propensity to trust machines (MTP). The predictive validity of the TIAS was reflected in Hypothesis 4—that trust measured on this scale would positively predict intentions to use each AI application.

#### 3.1.1 Method

##### 3.1.1.1 Participants

Two hundred and seventy US-based participants were recruited from Prolific (Palan and Schitter, 2018) in January 2023, comprising 136 men (50.2%), 128 women (47.4%), and 6 indicating another gender identity (2.2%). Participants’ ages ranged from 18 to 79 years, with a mean age of 34.9 years (*SD* = 13.17). Participants were compensated a pro-rated amount of £6.60 per hour.

##### 3.1.1.2 Materials and procedure

Participants recruited from Prolific were directed to the survey hosted on the Qualtrics platform. They first reviewed a participant information statement and provided consent to participate in the study. All participants completed the MTP and HTP scales before being randomly allocated to see a vignette reflecting a single AI application in either a high- or low-performance condition. Across the participant pool, high and low performance versions of each AI application were evenly presented. After reviewing the vignette, participants completed the TIAS and responded to the BI items. Finally, participants provided demographic information including age, gender, and level of education.

###### 3.1.1.2.1 Vignettes

Vignettes were drafted by the authors and designed to be written in accessible language and, as far as possible, comparable in length across conditions. An example vignette is provided in Table 2, and the full set is available in the Supplementary materials. A pilot study (see Supplementary materials) confirmed that the conditions described were perceived by participants to involve an element of risk, a necessary precondition for trust (Costa et al., 2018), and that they successfully manipulated perceived performance and integrity.

**Table 2:** Examples of the vignettes presented to participants for each trustworthiness condition.

Study/condition	Validation Study 1	Validation Study 2
	Performance	Integrity
	*All participants see the following vignette stem:* You are looking to get a new long-term phone plan. You go to a plan comparison website, Cell Select. The AI virtual assistant chats to you about your needs so it can help you find a plan. You have used this service before for a 6-month plan.	
Low	Last time, the AI assistant showed you a list of plans and you chose the top one. You later found out the plan had poor reception in your area. It also did not have enough data to cover your monthly internet usage. You were stuck in the contract for 6 months.	Last time, the AI assistant showed you plans from their premium providers (companies that have paid to be shown higher on the list). The actual best plans for you were on the second page. You were not told that the first results were sponsored and ended up choosing the first listed plan. This was more expensive than you needed.
High	When the AI assistant helped you last time, it showed you the best plans for you. You chose the top-listed plan and were happy with it.	Last time, the AI assistant showed you the best plans for you in a list. The AI recommendation was not affected by sponsorship, and all products were rated based on their quality. The best match was at the top of the list, and you chose it.

###### 3.1.1.2.2 Trust

Trust in the system presented in the vignette was measured using the 12-item Trust in Automation Scale (Jian et al., 2000). Items are designed to assess human trust in specific automated systems (see Table 1) and are rated by participants on a 7-point Likert scale where 1 = *Not at all* and 7 = *Extremely*. A trust score for each participant was derived from the mean of the 12-item ratings (Cronbach’s *α* = 0.94).

###### 3.1.1.2.3 Human trust propensity (HTP)

An individual’s general tendency to trust other humans was measured using a 4-item scale developed by Frazier et al. (2013). Each item (e.g., “My tendency to trust others is high”) was rated on a 7-point Likert scale where 1 = *Strongly disagree* and 7 = *Strongly agree*. An overall HTP score was calculated from the mean of item responses (Cronbach’s α = 0.93).

###### 3.1.1.2.4 Machine trust propensity (MTP)

A dispositional tendency to trust machines was measured on a 6-item scale developed by Merritt et al. (2013). Each item (e.g., “My tendency to trust machines is high”) was rated by participants on a 7-point Likert scale (where 1 = *Strongly disagree* and 7 = *Strongly agree*). An overall MTP score was calculated from the mean of item responses (Cronbach’s *α* = 0.90).

###### 3.1.1.2.5 Behavioral intention (BI)

Participants’ intentions to use the systems depicted in the vignettes were measured using three items designed to capture self-prediction (e.g., “I expect that I would follow the system’s recommendation.”), desire/forethought (e.g., “I would follow the system’s recommendation.”), and intent (e.g., “I would like to follow the system’s recommendation in this situation.”). The scale items were based on components of the theory of planned behavior, which formalizes the prediction of human behavior and asserts that behaviors are immediately determined by intentions (Armitage and Conner, 2001). Participants responded to each item on a 7-point Likert scale (where 1 = *Strongly Disagree* and 7 = *Strongly Agree*). An overall BI score was calculated from the mean of item responses (Cronbach’s *α* = 0.97).

#### 3.1.2 Results and discussion

Means and standard deviations for all AI applications across each performance condition are provided in Table 3.

**Table 3:** Means and standard deviations of all variables by AI application and condition: Study 1.

Variables	Self-driving car			Virtual assistant			Medical diagnosis app		
	Total	Low	High	Total	Low	High	Total	Low	High
Trust	3.73 (1.27)	2.95 (0.79)	4.55 (1.17)	4.20 (1.19)	3.38 (0.85)	5.03 (0.87)	3.6 (1.44)	2.66 (1.05)	4.66 (1.03)
HTP	4.55 (1.36)	4.53 (1.26)	4.57 (1.46)	4.24 (1.49)	4.40 (1.41)	4.09 (1.56)	4.23 (1.60)	4.28 (1.54)	4.18 (1.68)
MTP	4.84 (1.06)	5.00 (0.87)	4.67 (1.22)	4.89 (1.00)	4.95 (0.84)	4.82 (1.14)	4.64 (1.12)	4.67 (1.13)	4.61 (1.11)
BI	3.55 (1.75)	2.90 (1.44)	4.23 (1.88)	3.93 (1.75)	2.82 (1.53)	5.04 (1.15)	3.65 (1.99)	2.10 (1.52)	5.21 (0.85)
*N*	90	46	44	90	45	45	90	45	45

HTP, Human trust propensity; MTP, Machine trust propensity, BI, Behavioral intention.

##### 3.1.2.1 Hypotheses 1a and 1b: sensitivity to manipulations of trustworthiness (performance)

One-tailed independent samples *t*-tests indicated that reported trust measured by the TIAS was significantly higher in the high- than low-performance condition for all three AI applications self-driving car, *t*(74.87) = 7.54, *p* < 0.001, *d* = 1.6, 95%; virtual assistant, *t*(87.97) = 9.09, *p* < 0.001, *d* = 1.9, 95%; medical diagnosis app, *t*(87.98) = 9.11, *p* < 0.001, *d* = 1.9, 95%. These findings provide support for both Hypothesis 1a and 1b, showing that trust measured by the TIAS is sensitive to system performance, and that this sensitivity generalizes across a range of AI applications.

##### 3.1.2.2 Hypotheses 2 and 3: convergent validity

Pearson’s correlation coefficients were calculated to test convergent validity between trust measured by the TIAS and by both HTP and MTP. As can be seen in Table 4, HTP was not significantly correlated with trust for any of the AI applications. This holds when correlations were examined and broken down by performance condition, indicating a lack of support for Hypothesis 2. Hypothesis 3 was supported with MTP consistently related to trust under high-performance conditions.

**Table 4:** Pearson’s correlation coefficient between listed variables and TIAS scores: Study 1.

Variables	Self-driving car			Virtual assistant			Medical Diagnosis app		
	Total	Low	High	Total	Low	High	Total	Low	High
HTP	0.15	0.03	0.27	0.02	0.04	0.21	0.06	0.27	0.23
MTP	0.28**	0.15	0.67**	0.16	0.13	0.41**	0.13	−0.09	0.53**
*N*	90	46	44	90	45	45	90	45	45

** *p* < 0.001.

##### 3.1.2.3 Hypothesis 4: predictive validity

A hierarchical multiple regression was run for each application to assess whether TIAS scores are an independent positive predictor of BI over and above other predictor variables. For each AI application, BI was regressed on a five-step model in which demographic data was first entered, followed by MTP, HTP, trustworthiness condition, and TIAS score. Gender was coded as a dichotomous variable where woman = 1 and man = 2. Due to the low number of participants expressing an alternate gender identity (*n* = 6), this was not included as a predictor variable for the regression. For each model, assumptions of regression were met, including normality of residuals, independence of errors, linearity, and homoscedasticity.The first model, including only demographic predictors (gender, education, and age), was not statistically significant for any of the three AI applications (Table 5). The second model, including MTP as a predictor, was significant only for the self-driving car, *F*(4, 83) = 3.32, *p* = 0.01. The introduction of HTP in Model 3 similarly only explained a significant amount of variance in BI for the self-driving car, *F*(5, 82) = 3.24, *p* = 0.01, but not the virtual assistant or medical diagnostic app. The fourth model, including condition (low or high performance), significantly predicted BI for all applications (self-driving car, *F*(6, 81) = 7.32, *p* < 0.001; virtual assistant, *F*(6,82) = 10.08, *p* < 0.001; medical diagnostic app, *F*(6, 80) = 23.83, *p* < 0.001). The final model included trust scores and explained more than 70% of the variance in behavioral intentions for all applications (self-driving car, *F*(7,81) = 26.41, *p* < 0.001; virtual assistant, *F*(7, 81) = 31.73, *p* < 0.001; medical diagnostic app, *F*(7, 79) = 80.14, *p* < 0.001). Trust scores were the only significant predictor in this final model.

**Table 5:** Hierarchical multiple regression of intention to use AI applications: Study 1.

Models	*R*^2^			*B*			*ß*		
	Car	Ass’t	Med	Car	Ass’t	Med	Car	Ass’t	Med
Model 1	0.001	0.01	0.03						
Gender				0.37	0.16	0.14	0.10	0.04	0.03
Education				−0.01	−0.09	0.19	−0.01	−0.07	0.12
Age				0.01	−0.00	0.01	0.05	−0.02	0.09
Model 2	0.14**	0.01	0.04						
Gender				0.22	0.13	0.14	0.06	0.04	0.03
Education				−0.09	−0.09	0.19	−0.06	−0.07	0.12
Age				0.00	−0.00	0.01	0.02	−0.02	0.09
MTP				0.62	0.04	0.14	0.36**	0.02	0.08
Model 3	0.16**	0.01	0.04						
Gender				0.21	0.14	0.14	0.06	0.04	0.03
Education				−0.08	−0.09	0.18	−0.06	−0.07	0.12
Age				−0.00	−0.00	0.01	−0.00	−0.02	0.08
MTP				0.51	0.06	0.10	0.30*	0.04	0.06
HTP				0.24	−0.04	0.08	0.18	−0.04	0.07
Model 4	0.35**	0.42**	0.64**						
Gender				0.16	−0.10	0.24	0.04	−0.03	0.06
Education				−0.02	−0.16	0.01	−0.02	−0.11	0.01
Age				−0.00	0.01	0.01	−0.02	0.09	0.05
MTP				0.63	0.13	0.10	0.36**	0.08	0.05
HTP				0.21	0.04	0.15	0.16	0.03	0.12
Condition				0.16	−2.30	−3.10	−0.44**	−0.67**	−0.78**
Model 5	0.77**	0.73**	0.88**						
Gender				−1.58	0.28	0.06	0.01	0.08	0.01
Education				0.04	−0.02	0.04	−0.07	−0.01	0.03
Age				−0.10	−0.00	−0.00	−0.07	−0.03	−0.01
MTP				−0.01	−0.23	−0.07	−0.01	−0.13	−0.04
HTP				−0.01	−0.00	0.02	0.18*	−0.00	0.02
Condition				0.24	−0.14	−1.14	0.22*	−0.04	−0.29**
TIAS				0.79	1.23	0.99	0.96**	0.84**	0.71**
*N*	88	89	87						

Asterisks denoting statistical significance for R^2^ refer to the statistical significance of the change in R^2^ from the previous step. Gender: Woman = 1, Man = 2. Condition: 1 = low performance, 2 = high performance. *p < 0.05, **p < 0.001.

##### 3.1.2.4 Exploratory factor analysis

An exploratory factor analysis was conducted on the TIAS items using an oblique rotation method and 25 iterations. Inspection of a scree plot and eigenvalues converged on a two-factor structure. Factor 1 explained 71% of the variance and comprised items 1, 2, 3, and 5 with loadings between 0.54 and 0.84. Factor 2 explained 29% of the variance and consisted of items 6 to 12, with loadings between 0.53 and 0.93. Item 4 loaded on factors 1 and 2, with loadings of 0.40 and 0.48, respectively.

In Study 1, we conducted tests to validate the TIAS scale by examining its convergent and predictive validity across three AI applications. We found that trust scores were sensitive to manipulations of system trustworthiness, with participants reporting significantly less trust in systems that exhibited lower performance. Contrary to our expectations, trust, as measured by the TIAS, was not positively correlated with HTP but showed consistent positive correlations with MTP across the three AI applications. Supporting the predictive validity of the TIAS, trust scores uniquely predicted intentions to use the AI applications.

### 3.2 Study 2: integrity

In Study 2, between-subjects conditions manipulated the integrity of the system, rather than its performance. We also substituted a flight booking app for the medical app to permit more ecologically valid variations in integrity. Hypotheses probed the same aspects of scale validity tested in Study 1. Hypothesis 5a predicted that participants in high integrity conditions would report significantly higher levels of trust than those in the low integrity condition, while Hypothesis 5b anticipated that this result would generalize across three AI applications. Hypotheses 6 and 7 tested expectations of a significant positive association between the TIAS and both HTP (Hypothesis 6) and MTP (Hypothesis 7). Our final hypothesis was that trust would positively predict intentions to use each AI application (Hypothesis 8).

#### 3.2.1 Method

Participants. Two hundred and seventy US-based participants were recruited from Prolific in January 2023. They comprised 135 men (50%), 130 women (48.1%), and five indicating another gender identity (1.9%), with ages ranging from 18 to 77 years (*M* = 37.43, *SD* = 13.83). Participants were compensated £1.10 (pro-rata of £6.60 per hour).

#### 3.2.2 Materials and procedure

Materials and procedure were as reported in Study 1. Cronbach’s *α* coefficients for measured variables in this study were as follows: Trust (*α* = 0.94), HTP (*α* = 0.93), MTP (*α* = 0.91), and BI (*α* = 0.97).

#### 3.2.3 Results and discussion

Means and standard deviations for all AI applications across each integrity condition are provided in Table 6.

**Table 6:** Means and standard deviations of all variables by AI application and condition: Study 2.

Variables	Self-driving car			Virtual assistant			Airline booking app		
	Total	Low	High	Total	Low	High	Total	Low	High
Trust	3.90 (1.40)	3.03 (1.10)	4.78 (1.08)	3.97 (1.45)	2.83 (0.98)	5.09 (0.84)	4.00 (1.36)	3.18 1.32	4.83 (0.77)
HTP	4.07 (1.48)	4.23 (1.49)	3.92 (1.47)	4.48 (1.57)	4.65 (1.25)	4.72 (1.00)	4.47 (1.48)	4.49 (1.50)	4.45 (1.47)
MTP	4.65 (0.98)	4.68 (1.08)	4.62 (0.89)	4.84 (1.22)	4.74 (1.27)	4.94 (1.16)	4.69 (1.13)	4.65 (1.25)	4.72 (1.00)
BI	4.22 (1.84)	3.42 (1.71)	5.04 (1.62)	4.08 (1.69)	2.92 (1.51)	5.22 (0.91)	4.18 (1.95)	2.93 (1.80)	5.44 (1.12)
*N*	91	46	45	89	44	45	90	45	45

HTP, Human trust propensity; MTP, Machine trust propensity; BI, Behavioral intention. For all scales, higher scores indicate a higher level of the construct.

##### 3.2.3.1 Hypotheses 5a and 5b: sensitivity to trustworthiness manipulations (integrity)

One-tailed independent samples t-tests indicated that TIAS scores were significantly higher in the high than low integrity condition for all three AI applications (self-driving car, *t*(89) = 7.68, *p* < 0.001, *d* = 1.61, 95%; virtual assistant, *t*(84.16) = 11.67, *p* < 0.001, *d* = 2.48, 95%; airline booking, *t*(70.76) = 7.23, *p* < 0.001, *d* = 1.5, 95%). These findings indicate that the TIAS is sensitive to manipulations of system integrity, and this sensitivity generalizes across a range of AI applications.

##### 3.2.3.2 Hypotheses 6 and 7: convergent validity

To test the convergent validity between scores on the TIAS and both HTP and MTP, we calculated Pearson’s correlation coefficients (Table 7). The hypothesized positive association between trust and HTP was only observed for the virtual assistant. However, when broken down by condition, we also observed a significant positive correlation between TIAS scores and HTP in the high integrity condition for both the virtual assistant and the airline booking application, providing partial support for Hypothesis 6. Hypothesis 7 was supported by significant positive associations observed between MTP and trust scores for all applications.

**Table 7:** Pearson’s correlation coefficient between listed variables and TIAS scores: Study 2.

Variables	Self-driving car			Virtual assistant			Airline booking app		
	Total	Low	High	Total	Low	High	Total	Low	High
HTP	0.09	−0.03	−0.02	0.26*	0.01	0.30*	0.12	0.07	0.35*
MTP	0.24*	0.14	0.59**	0.29**	0.31*	0.43*	0.37**	0.45*	0.46*
*N*	91	46	45	89	44	45	90	45	45

*p < 0.05, **p < 0.001.

##### 3.2.3.3 Hypothesis 8: predictive validity

As with Study 1, a five-step hierarchical multiple regression was conducted with BI as the dependent variable for each AI application (self-driving car, virtual assistant, and medical diagnosis app). Demographic variables were entered into the model first, followed by MTP, HTP, and TIAS scores. Gender was entered as a dichotomous variable where woman = 1 and man = 2.As with Study 1, the model, including only gender, age, and education, did not significantly predict intentions to use any of the AI applications (Table 8). The introduction of MTP and HTP in Models 2 and 3 were only statistically significant for the virtual assistant, Model 2, *F*(4,82) = 3.36, *p* = 0.01; Model 3, *F*(5,81) = 3.41, *p* = 0.01. The fourth model, including integrity condition as a predictor, was significant for all applications and explained between 30 and 55% of BI (self-driving car, *F*(6,82) = 6.53, *p* < 0.001; virtual assistant, *F*(6,80) = 15.36, *p* < 0.001; airline booking, *F*(6,82) = 13.18, *p* < 0.001). The final model, including trust scores, was also significant for all applications and explained more than 70% of the variance in intentions to use all of the tested AI systems (self-driving car, *F*(7,81) = 26.41, *p* < 0.001; virtual assistant, *F*(7,79) = 31.47, *p* < 0.001; airline booking, *F*(7,81) = 46.06, *p* < 0.001).

**Table 8:** Hierarchical multiple regression of intention to use AI applications: Study 2.

Models	*R*^2^			*B*			*ß*		
	Car	Ass’t	Air	Car	Ass’t	Air	Car	Ass’t	Air
Model 1	0.05	0.05	0.02						
Gender				0.68	−0.49	0.10	0.18	−0.14	0.03
Education				0.05	−0.23	0.02	0.03	−0.18	0.01
Age				−0.01	−0.01	−0.02	−0.09	−0.05	−0.13
Model 2	0.10**	0.14**	0.09**						
Gender				0.70	−0.59	0.07	0.19	−0.18	0.02
Education				0.04	−0.25	−0.01	0.03	−0.19	−0.01
Age				−0.01	−0.01	−0.01	−0.12	−0.11	−0.10
MTP				0.41	0.41	0.47	0.22*	0.30*	0.27*
Model 3	0.11	0.17**	0.09						
Gender				0.75	−0.51	0.07	0.20	−0.15	0.02
Education				0.04	−0.19	−0.01	0.03	−0.15	−0.01
Age				−0.01	−0.01	−0.01	−0.10	−0.12	−0.10
MTP				0.47	0.37	0.48	0.25*	0.26*	0.27*
HTP				−0.13	0.20	−0.01	−0.10	0.19	−0.01
Model 4	0.32**	0.54**	0.49**						
Gender				0.96	−0.38	−0.05	0.26*	−0.11	−0.01
Education				−0.11	−0.15	0.05	−0.08	−0.12	0.03
Age				−0.01	−0.01	−0.01	−0.08	−0.08	−0.07
MTP				0.47	0.31	0.46	0.25*	0.23*	0.27*
HTP				−0.07	0.06	−0.00	−0.06	0.05	−0.00
Condition				−1.80	−2.09	−2.47	−0.47**	−0.62**	−0.64**
Model 5	0.70**	0.74**	0.80**						
Gender				0.25	−0.06	−0.12	0.07	−0.02	−0.03
Education				0.07	0.01	0.08	0.05	0.01	0.05
Age				−0.00	−0.00	0.00	−0.01	−0.01	0.02
MTP				−0.11	0.02	0.00	−0.06	0.01	0.00
HTP				0.06	0.04	−0.05	0.05	0.04	−0.04
Condition				0.50	−0.11	−0.66	0.13	−0.03	−0.17*
TIAS				1.21	0.95	1.14	0.91**	0.82**	0.79**
*N*	89	87	89						

Asterisks denoting statistical significance for R^2^ refer to the statistical significance of the change in R^2^ from the previous step. Gender: woman = 1, man = 2. Condition: low integrity = 1, high integrity = 2. *p < 0.05, **p < 0.001.

##### 3.2.3.4 Confirmatory factor analysis

We conducted a confirmatory factor analysis of the two-factor structure observed in the exploratory factor analysis in Study 1. As item 4 was loaded on both factors in Study 1, we compared two models. Model 1 loaded item 4 on Factor 1 with items 1, 2, 3, and 5, and Factor 2 comprised items 6–12. In Model 2, item 4 was instead loaded on Factor 2 with items 6–12, and Factor 1 comprised only items 1, 2, 3, and 5. The analysis was conducted in R using the lavaan package. Fit indices for each model are given in Table 9.

**Table 9:** Goodness-of-fit indices for TIAS factor models: Study 2.

Fit indices	Model 1	Model 2
*Χ*^2^	156.38**	224.86**
CFI	0.96	0.94
TLI	0.95	0.92
RMSEA	0.09	0.11
AIC	10257.12	10325.60

*p < 0.05, **p < 0.001.

Both factor models demonstrate adequate to good fit according to the cutoffs provided by Hu and Bentler (1999) (*RMSEA* < 0.06 = good, *CFI* > 0.95 = good, *TLI* > 0.95 = good). Model 1, in which item 4 is included in Factor 1, is shown by all indices as the better-fitting model. This is consistent with the item loadings reported by Spain et al. (2008) and Perrig et al. (2023). Factor loadings of the two factors in Model 1 are given in Supplementary materials.

In Study 2, we extended our findings by testing the sensitivity of scores on the TIAS to manipulations of integrity rather than performance. Once again, participants reported significantly less trust in systems that were shown to be less trustworthy. Consistent with Study 1, trust, as measured by the TIAS, was not consistently positively correlated with HTP but did show positive correlations with MTP. TIAS scores also uniquely predict intentions to use AI applications.

## 4 Development and validation of the Short Trust in Automation Scale (S-TIAS)

In this section, we report on the development and validation of a time-effective yet psychometrically rigorous short form of the scale. To develop the S-TIAS, item analysis was conducted on data collected in Studies 1 and 2. The 12 items of the full scale were compared on four parameters for each AI application within each study: (i) correlation of the item with the total test score (scored without item), (ii) Cronbach’s *α* of the full scale when that item is dropped, (iii) average inter-item correlation when that item is dropped, and (iv) correlation of the item with the BI criterion measure. Table 10 provides a summary of item statistics for each parameter across the three AI applications in each of the two studies (full item statistics are provided in Supplementary materials). Higher performing items are those with (1) higher correlations with the total test score, (2) lower Cronbach’s α and average inter-item correlations when the item is omitted from the scale, and (3) higher correlations with the BI criterion.

**Table 10:** Summary of item analysis parameters showing range of statistics across applications and studies.

TIAS Item	Corr. with total test score	Cronbach’s α when item dropped	Average inter-item corr. when item dropped	Corr. of item with BI criterion measure
1	0.62–0. 89	0.92–0.94	0.50–0.60	0.53–0.80
2	0.46–0.76	0.92–0.95	0.52–0.62	0.31–0.73
3	0.68–0.81	0.92–0.94	0.50–0.59	0.55–0.74
4	0.71–0.86	0.92–0.94	0.49–0.59	0.63–0.79
5	0.57–0.74	0.92–0.94	0.52–0.61	0.43–0.68
6	0.83–0.93	0.91–0.94	0.48–0.58	0.77–0.89
7	0.54–0.82	0.92–0.94	0.52–0.60	0.59–0.81
8	0.63–0.78	0.92–0.94	0.51–0.60	0.53–0.78
9	0.75–0.90	0.91–0.94	0.48–0.59	0.74–0.92
10	0.76–0.88	0.91–0.94	0.48–0.59	0.71–0.92
11	0.84–0.91	0.91–0.94	0.48–0.57	0.81–0.92
12	0.33–0.39	0.93–0.95	0.56–0.65	0.29–0.42

The four most strongly performing items were identified for all AI applications across both studies. There was considerable convergence across all parameters, with TIAS-11 (“I can trust the AI assistant”), TIAS-6 (“I am confident in the AI assistant”) and TIAS-10 (“The AI assistant is reliable”) most frequently represented among the strongest performing items. We note that the integrity dimension of trustworthiness is not represented among these items, suggesting that performance is primary in users’ evaluation of trust in AI. To validate the S-TIAS, we manipulated the AI system’s performance in Study 3, while in Study 4, we manipulated its integrity or fairness. Within each study, we used a 2 (trustworthiness condition: high, low) x 2 (trust scale version: TIAS, S-TIAS) between-subjects design.

### 4.1 Study 3: validation of the S-TIAS (performance)

#### 4.1.1 Method

##### 4.1.1.1 Participants

Participants were based in the United States and recruited from the Prolific platform in October 2023. All participants were compensated £1.10 for their time. Data were collected from 182 participants; 88 (48.4%) women, 87 (47.8%) men, six participants (3.3%) who reported another gender identity, and one (0.5%) who preferred not to provide gender information. Participants’ ages ranged from 18 to 73 years, with a mean age of 37.56 (*SD* = 12.92).

##### 4.1.1.2 Materials and procedure

###### 4.1.1.2.1 Vignettes

Trustworthiness (performance) was manipulated using the vignettes in Table 2.

###### 4.1.1.2.2 Trust in Automation Scale (TIAS)

Items in the 12-item TIAS (Jian et al., 2000) were rated by participants on a 7-point scale where 1 = *Not at all* and 7 = *Extremely*. Responses on the 12 items are averaged to provide an overall trust score (Cronbach’s *α* = 0.95).

###### 4.1.1.2.3 Short Trust in Automation Scale (S-TIAS)

The three items that form the S-TIAS are “I am confident in the AI assistant,” “The AI assistant is reliable,” and “I can trust the AI assistant.” Responses are made on a 7-point Likert scale where 1 = Not at all and 7 = Extremely, and outcomes on the three items are averaged to yield an overall trust score (Cronbach’s *α* = 0.97).

HTP (Cronbach’s *α* = 0.88), MTP (Cronbach’s *α* = 0.91), and BI (Cronbach’s *α* = 0.96) were measured using the same materials reported in Studies 1 and 2.

#### 4.1.2 Results

The TIAS and the S-TIAS each showed excellent internal consistency, indicating comparable reliability of measurement with both versions.

##### 4.1.2.1 Sensitivity to differences in trustworthiness

Independent samples t-tests were conducted to test the sensitivity of each trust measure to manipulations of the trustworthiness of the AI system. Self-reported trust was significantly higher in the high-performance than low-performance condition when measured on both scales (Table 11). From this, it can be inferred that the S-TIAS is sufficiently sensitive and valid for identifying the effects of performance as a key antecedent factor of trust.

**Table 11:** Means, standard deviations, and t-test outcomes for the TIAS and S-TIAS: Study 3.

Measure	*M* (*SD*): Low trustworthiness	*M* (*SD*):High trustworthiness	*t*	*df*	Cohen’s *d*
TIAS	3.45 (0.99)	5.05 (1.08)	7.41***	87.78	1.6
S-TIAS	2.66 (1.37)	5.22 (1.09)	9.89***	83.84	2.1

***p < 0.001.

##### 4.1.2.2 Convergent validity

Consistent with findings from Study 1, trust measured using the TIAS did not demonstrate positive associations with HTP but was positively correlated with MTP. Although showing somewhat smaller correlations with MTP, a similar pattern of results emerged when measuring trust using the short-form scale, suggesting both forms of the scale have comparable convergent validity (Table 12).

**Table 12:** Pearson’s correlation coefficient between listed variables and TIAS and S-TIAS scores: Study 3.

Variables	TIAS			S-TIAS		
	Total	Low	High	Total	Low	High
HTP	−0.01	0.02	0.08	−0.02	0.14	0.11
MTP	0.58**	0.47**	0.73**	0.31*	0.31	0.54**
*N*	91	46	45	91	45	46

* p < 0.01, ** p < 0.001.

##### 4.1.2.3 Predictive validity

Two hierarchical multiple regression analyses were conducted to assess whether the scores on the S-TIAS predicted BI similarly to the TIAS. For all analyses, predictor variables were entered in the order of age, education, gender (where woman = 1 and man = 2), HTP, MTP, trustworthiness condition (low = 0, high = 1), and trust score. As with previous studies, participants who did not identify with a binary gender identity were excluded from the analysis.

Table 13 provides standardized coefficients, the amount of variance in BI accounted for by each model (*R*^2^), and the degree and statistical significance of increases in explained variance between models (*R*^2^ change) for analyses conducted with the S-TIAS. Table 14 provides the same information for analyses conducted with the TIAS.

**Table 13:** Hierarchical multiple regression predicting behavioral intention using the S-TIAS: Study 3.

Predictors	Model 1	Model 2	Model 3	Model 4	Model 5
	*ß*	*ß*	*ß*	*ß*	*ß*
Age	0.03	0.03	0.02	−0.05	−0.04
Education	−0.10	−0.10	−0.09	0.04	0.10
Gender	0.08	0.09	0.04	−0.07	−0.01
HTP		−0.03	−0.08	0.04	−0.01
MTP			0.32**	0.27***	0.06
Condition				0.75***	0.22**
S-TIAS					0.75***
*N*	89	89	89	89	89
*R*^2^	0.02	0.02	0.12	0.63	0.85
*R*^2^ change		0.001	0.10**	0.51***	0.23***

Gender: Woman = 1, Man = 2, HTP = human trust propensity, MTP = machine trust propensity, Condition: 0 = low performance, 1 = high performance, *p < 0.05, **p < 0.01, ***p < 0.001.

**Table 14:** Hierarchical multiple regression predicting behavioral intention using the TIAS: Study 3.

Predictors	Model 1	Model 2	Model 3	Model 4	Model 5
	*ß*	*ß*	*ß*	*ß*	*ß*
Age	0.15	0.16	0.17	0.08	0.02
Education	0.01	0.02	−0.01	−0.06	−0.03
Gender	0.13	0.13	0.06	0.03	0.03
HTP		−0.05	−0.07	−0.004	0.03
MTP			0.39***	0.27**	−0.09
Condition				0.62***	0.16*
TIAS					0.83***
*N*	86	86	86	86	86
*R*^2^	0.04	0.04	0.18	0.54	0.80
*R*^2^ change		0.002	0.14***	0.36***	0.27***

Gender: Woman = 1, Man = 2, HTP = human trust propensity, MTP = machine trust propensity, Condition: 0 = low performance, 1 = high performance, *p < 0.05, **p < 0.01, ***p < 0.001.

The final model with all variables entered shows that trust measured on S-TIAS is a significant predictor of intention to use the virtual AI assistant. The beta coefficient of 0.75 is comparable to the beta coefficient of 0.83 observed in the identical regression conducted using the full TIAS. Further supporting the predictive validity of the S-TIAS, the significant *R*^2^ change value for Model 5 indicates that trust explains variance in BI over and above other variables. The comparable *R*^2^ value for the TIAS is 0.27.

### 4.2 Study 4: validation of the S-TIAS (integrity)

#### 4.2.1 Method

##### 4.2.1.1 Participants

Study 4 comprised 180 participants, 79 (43.9%) of whom were women, 97 (53.9%) men, and 4 (2.3%) identified as an alternate gender. Participants were aged between 19 and 75, with a mean age of 38.03 years (*SD* = 12.16). Data were collected from Prolific in October 2023.

##### 4.2.1.2 Materials and procedure

Materials and procedure were as reported in Study 3. Cronbach’s *α* coefficients for measured variables in this study were TIAS (*α* = 0.95), S-TIAS (*α* = 0.97), HTP (*α* = 0.98), MTP (*α* = 0.90), and BI (*α* = 0.97).

#### 4.2.2 Results

##### 4.2.2.1 Sensitivity to differences in trustworthiness

Independent samples t-tests showed that both the full scale and short form recorded significantly higher trust in the high integrity condition (Table 15).

**Table 15:** Means, standard deviations, and t-test outcomes for the TIAS and S-TIAS: Study 4.

Measure	*M* (*SD*) – Low trustworthiness	*M* (*SD*) – High trustworthiness	*t*	*df*	Cohen’s *d*
TIAS	3.19 (0.97)	4.79 (0.85)	8.32***	86.39	1.8
S-TIAS	2.92 (1.48)	5.30 (0.99)	8.89***	74	1.9

***p < 0.001.

##### 4.2.2.2 Convergent validity

In Study 4, we again observe a lack of association between HTP and trust measured on either the TIAS or the S-TIAS (Table 16). There is, however, a similar pattern of positive correlations between both scales and MTP.

**Table 16:** Pearson’s correlation coefficient between listed variables and TIAS and S-TIAS scores: Study 4.

Variables	TIAS			S-TIAS		
	Total	Low	High	Total	Low	High
HTP	0.14	0.18	0.22	0.14	0.07	0.22
MTP	0.23*	0.19	0.63***	0.24*	0.21	0.55***
*N*	90	45	45	89	44	45

*p < 0.05, **p < 0.01, ***p < 0.001.

##### 4.2.2.3 Predictive validity

Hierarchical regressions on data collected in Study 4 show a similar pattern of results as those for Study 3. Tables 17, 18 provide beta coefficients and values of *R*^2^ and *R*^2^ change for analyses conducted with the S-TIAS and TIAS, respectively.

**Table 17:** Hierarchical multiple regression predicting behavioral intention using S-TIAS: Study 4.

Predictors	Model 1	Model 2	Model 3	Model 4	Model 5
	*ß*	*ß*	*ß*	*ß*	*ß*
Age	0.05	0.06	0.06	−0.02	0.03
Education	−0.03	−0.04	−0.08	0.01	−0.01
Gender	0.01	0.01	0.04	0.06	0.05
HTP		0.13	0.04	−0.03	−0.01
MTP			0.24*	0.25**	0.000
Condition				0.68***	0.03
S-TIAS					0.92***
*N*	88	88	88	88	88
*R*^2^	0.003	0.02	0.07	0.51	0.88
*R*^2^ change		0.02	0.05*	0.44***	0.37***

Gender: Woman = 1, Man = 2, HTP = human trust propensity, MTP = machine trust propensity, Condition: 0 = low integrity, 1 = high integrity, *p < 0.05, **p < 0.01, ***p < 0.001.

**Table 18:** Hierarchical multiple regression predicting behavioral intention using TIAS: Study 4.

Predictors	Model 1	Model 2	Model 3	Model 4	Model 5
	*ß*	*ß*	*ß*	*ß*	*ß*
Age	−0.04	−0.05	−0.04	0.02	0.03
Education	−0.03	−0.03	0.002	−0.003	0.04
Gender	−0.11	−0.09	−0.09	−0.07	−0.11
HTP		0.16	0.11	0.12	0.05
MTP			0.17	0.24**	0.07
Condition				0.72***	0.25**
TIAS					0.66***
*N*	88	88	88	88	88
*R*^2^	0.02	0.04	0.07	0.58	0.76
*R*^2^ change		0.03	0.03	0.51***	0.19***

Gender: Woman = 1, Man = 2, HTP = human trust propensity, MTP = machine trust propensity, Condition: 0 = low integrity, 1 = high integrity, *p < 0.05, **p < 0.01, ***p < 0.001.

Once again, the S-TIAS shows validity in predicting intentions to use the virtual AI assistant with a beta coefficient that exceeds that of the TIAS in an equivalent model. Trust measured with the S-TIAS also explains a significant degree of variance in BI over and above other relevant constructs, and to a greater extent than the TIAS.

## 5 General discussion

The first purpose of this research was to test the validity of the commonly used Trust in Automation Scale (Jian et al., 2000) for measuring trust in different types of contemporary AI-based applications. The second purpose of the research was to extract a short form of the TIAS and subject it to psychometric validation.

We first investigated whether differences in the trustworthiness of systems led to differences in trust as measured by the TIAS. In Study 1 and Study 2, we observed significantly higher trust reported in high performance and integrity conditions, suggesting that measurement of trust using the TIAS is sensitive to trustworthiness evaluations on multiple dimensions. Notably, this result was observed across a range of scenarios depicting different AI applications in each study, supporting the generalizability of the scale.

Investigations of the convergent validity of the scale yielded mixed results. Previous research has proposed that a general tendency to trust humans (Riedl, 2022) or machines (Faulhaber et al., 2021; Huang and Bashir, 2017) is a contributing factor in the formation of trust in a technological system. Consequently, we expected to see positive relationships between AI and both HTP and MTP. The expected relationship between MTP and TIAS was observed for all applications in Study 2, in which system integrity was manipulated. However, in Study 1, results were less clear, with the hypothesized correlations only consistently found in the high-performance condition. An explanation for these results may be the influence of a third variable, such as the Perfect Automation Schema (PAS) (Gibson et al., 2023). PAS is a cognitive schema that reflects an individual’s expectations of automated systems. It comprises two key factors: all-or-none thinking and high expectations. It may be that when an AI system underperforms (as in our manipulations), reported trust is more significantly influenced by a variable such as PAS than dispositional trust in machines (MTP). Overall, however, the relationships observed between MTP and TIAS scores were sufficiently consistent to demonstrate convergent validity.

We did not observe the same degree of consistency in the relationship between HTP and trust. For the most part, correlations between the two constructs were not statistically significant. This is a notable finding, as trust propensity is frequently included in models of trust in automation and AI (Hoff and Bashir, 2015; Lee and See, 2004), but it is not always specified within such models whether trust propensity refers to a general tendency to trust humans or machines. To the best of our knowledge, it has not been empirically established that the propensity to trust humans transfers to trust in AI. The present findings suggest that there may be qualitative or quantitative differences in the relationships between HTP and MTP and trust in AI that warrant further investigation.

Supporting the predictive validity of the scale, we found that across all applications in both studies, trust, as measured by the TIAS, was a significant predictor of intention to use the system. In each case, TIAS scores accounted for the greatest proportion of variance in BI to a significant degree. Nevertheless, we note that although measuring behavioral intent can provide useful insights into people’s trust, attitudes, and motivations, it has limitations compared to measuring actual behavior. People may not always act in accordance with their intentions, as their behavior may be influenced by external variables such as social influences and difficulty in performing the behavior (Armitage and Conner, 2001). An extension of this research to incorporate measures of actual behavior in human–AI interactions across a broader set of use cases would be valuable.

Our findings contribute to converging evidence of the TIAS measuring an oblique two-factor model of trust (Perrig et al., 2023; Spain et al., 2008). In Study 1, an exploratory factor analysis suggested a two-factor model, with factor loadings that may reflect the trust and distrust item clusters proposed by Jian et al. (2000). A confirmatory factor analysis conducted on the Study 2 data supported this two-factor model. We cannot, however, rule out alternative explanations, including that the factors are a methodological artifact of the distribution of reverse-scored items in the scale or that the factors each load on a second higher order trust construct. Nonetheless, given that the high internal consistency of the overall scale suggests the items are measuring a similar construct, we conclude that the TIAS is fit to measure the concept of trust in a range of AI systems.

In developing the S-TIAS, we extracted three high-performing items from the full TIAS. In Study 3 and Study 4, the S-TIAS demonstrated excellent internal consistency, supporting its reliability as a short measure of trust. Trust measured by the S-TIAS was significantly greater for the virtual AI assistant with high trustworthiness in terms of both performance and integrity than the comparable low trustworthiness conditions. These findings suggest that the S-TIAS is sensitive to trustworthiness evaluations on multiple dimensions. This is of particular note, given the composition of the short form. The full TIAS contains items intended to capture aspects of trust related to performance (e.g., “The system is reliable”) and integrity (e.g., “The system has integrity”). However, statistical analyses of the contribution of the latter items to the reliability and validity of the scale did not support their inclusion in the S-TIAS. Nonetheless, the results of Study 4 demonstrate that the S-TIAS has the same capacity to capture differences in trust in response to integrity violations as the full-length TIAS.

The S-TIAS also demonstrated good predictive validity. In Study 3 and Study 4, trust measured by the S-TIAS significantly predicted reported intentions to use the virtual AI assistant. The influence of trust on BI also distinguished it from other related constructs, including HTP and MTP. This pattern of results was consistent with measurements taken using the full 12-item TIAS. Studies that validate the S-TIAS using measures of actual behavior across a broader set of use cases would be valuable.

Many fundamental and applied studies exploring trust in AI will benefit from a quicker and yet still rigorous method of measurement (e.g., McGrath et al., 2024a). The S-TIAS is likely to be of great benefit to investigations of the sustained interactions with AI anticipated in many workplaces. There is a pressing need to understand the trajectories of trust development and maintenance in such environments (National Academies of Sciences, Engineering, and Medicine, 2022; McGrath et al., 2024b; O’Neill et al., 2023). To do so requires frequent, episodic capture of trust levels to identify and monitor change over time. The S-TIAS represents a psychometrically sound alternative to potentially less practical long-form scales or short *ad hoc* and untested trust measurement items. However, while the S-TIAS represents a gain in practicality over the 12-item TIAS, there will no doubt continue to be lab- and field-based experimental needs for even less disruptive measurement methods. In future studies, we will report on psychometric evaluation of the validity of a single-item self-report measure of trust.

Human trust is of considerable importance to researchers, developers, and users of AI because it predicts our willingness to use these technologies (Lee and See, 2004). The capacity to quantify trust becomes of even greater importance as we head into an era of increasing interest in human–AI teaming. Calibrated trust is relevant to a range of elements of human interaction with AI, including the role and outcomes of transparency (Zerilli et al., 2022) and explainability (Hoffman et al., 2023) and reliance on a system once adopted (Lee and See, 2004). Rigorous investigation of these relationships requires a reliable and valid measure of trust. While recognizing the limitations of self-report scales, such measures remain a practical means of assessing latent constructs in human data collection. Our results suggest that the TIAS, initially developed by Jian et al. (2000) for measuring trust in automation, may be a reliable and valid means of quantifying human trust in AI. We also find that the S-TIAS meets the criteria for validity independently and when compared to the TIAS, supporting its use as a practical and valid alternative for identifying antecedent factors of trust and predicting outcomes based on trust levels.

## Data Availability

The datasets presented in this study can be found in online repositories. The names of the repository/repositories and accession number(s) can be found at: https://data.csiro.au/collection/csiro:64559.
