# Supplementary material for: Measuring trust in artificial intelligence: validation of an established scale and its short form
Source: Front Artif Intell. 2025 May 9;8:1582880. doi: 10.3389/frai.2025.1582880 (PMC12098057; doi:10.3389/frai.2025.1582880)
Supplement: Supplementary file 1 [file Data_Sheet_1.pdf]

Supplementary Material

1 Vignette Set for each Trustworthiness Condition

Supplementary Table 1. Vignette Set for each Trustworthiness Condition

	Study 1. Performance	Study 2. Integrity
Autonomous Vehicle		
	All participants see the following vignette stem:<br>*You are running late to an important job interview and your normal form of transportation is unavailable. You can't get a lift using a regular rideshare, but you can get a new self-driving car that claims to be able to get you to the interview on time. You have used this once before to meet up with a friend.*	
High Condition	Last time you used the self-driving car, it drove safely and got you to your destination when it said it would.	Last time you used the self-driving car, it told you a price up front. After changes in traffic conditions, it managed to use less fuel than planned and gave you back the money it saved.
Low Condition	Last time you used the self-driving car, it made many sharp turns and stopped suddenly a few times. It dropped you off 10 minutes late and 3 blocks away from your destination.	Last time you used the self-driving car, it told you a price up front. When you arrived, you were charged triple the price because of surge pricing you were not told about.
Virtual Assistant		
	All participants see the following vignette stem:<br>*You are looking to get a new long-term phone plan. You go to a plan comparison website, Cell Select. The AI virtual assistant chats to you about your needs so it can help you find a plan. You have used this service before for a six month plan.*	
High Condition	When the AI assistant helped you last time, it showed the best plans for you. You chose the top listed plan and were happy with it.	Last time, the AI assistant showed you the best plans for you in a list. The best match was at the top of the list and you chose it. Unlike some other comparison companies, the AI system is not affected by sponsorship and all products are rated based on their quality.
Low Condition	Last time, the AI assistant showed you a list of plans and you chose the top one. You later found out the plan had poor reception in your area. It also did not have enough data to cover your monthly internet usage. You were stuck in the contract for six months.	Last time, the AI assistant showed you plans from their premium providers (more expensive companies that have paid to be shown higher on the list). The actual best plans for you were on a second page. You were not told that the first results were sponsored and ended up choosing the first

		listed plan. This was more expensive than you needed.
DataDoc Recommender system		
	All participants see the following vignette stem:<br>*You wake up in your hotel room while on an overseas holiday trip. You have a fever, rash and unusual pain in your stomach. You don't have access to your usual doctor, but do have access to an AI medical app called DataDoc. DataDoc has access to a large database of medical information. It checks your symptoms and gives you medical advice. You have used this once before when you were very dizzy and had a headache.*	
High Condition	Last time, DataDoc predicted it was most likely that you had an ear infection. It directed you to the correct medicine. You felt better in a few days.	
Low Condition	Last time, DataDoc predicted it was most likely that you had a broken leg. It told you that you needed surgery on your shoulder to get better. This caused you stress and left you feeling worse.	
Airline Profiling System		
	All participants see the following vignette stem:<br>*You are booking a holiday flight. You go online to a flight booking system that offers flights to your holiday destination. You have used this booking system before and found that the ticket and bag pricing just fell within your budget.*	
High Condition		Later on you found out that the booking AI works by checking how many seats there are and comparing prices across airlines. The AI makes sure that you pay the lowest price possible for the flight.
Low Condition		Later on you found out that the booking AI works by taking your internet, personal expense, and other social media history. This data is used to predict the highest amount you would be willing to pay. The AI system then only offers you tickets at that price.

2 Pilot Study

We conducted a pilot study to check three elements of our stimulus vignettes. The first was that the conditions described were perceived by participants to involve an element of risk. It has been argued that where there is no risk, there is no need for trust, and that an expectation of positive outcomes in the absence of vulnerability is better characterized as confidence than trust (Costa et al., 2018). We also wished to confirm that our vignettes successfully manipulate perceived performance (Study 1) and integrity (Study 2) across high and low conditions.

2.1 Method

2.1.1 Participants

Eighty participants resident in the United States were recruited from the online platform Prolific (Palan & Schitter, 2018). As Prolific is hosted in the United Kingdom, participants were paid a pro rata'd amount equivalent to £6.60 per hour (0.55p) to complete the study. Participants were 38 men (47.5%), 39 women (48.75%), and three individuals indicating an alternate gender identity (3.75%), ranging in age from 18 to 76 years (M = 37.59, SD = 15.22).

2.1.2 Materials

Vignettes. In this pilot study we tested the full range of vignettes intended for inclusion in Studies 1 and 2. These vignettes were drafted by the authors and designed to be written in accessible language and, as far as possible, to be comparable in length across conditions. Vignettes were drafted for four applications (self-driving car, virtual assistant recommending a mobile phone plan, a diagnostic medical app, and flight booking app) to investigate the generalizability of trust scores derived from the TIAS across a range of AI systems.

Perceived Risk was measured using a single self-report sliding scale ("There is risk in using this AI system") ranging from 0 to 100, where higher scores indicated greater agreement with the statement.

Perceived Performance was measured using a single self-report sliding scale ("The system performs well") ranging from 0 to 100, where higher scores indicated greater agreement with the statement.

Perceived Integrity was measured using a single self-report sliding scale ("The system is fair") ranging from 0 to 100, where higher scores indicated greater agreement with the statement.

2.1.3 Procedure

Participants recruited on Prolific were directed to the Qualtrics survey platform where they were presented with a participant information statement and gave their consent to take part in the study. As two of the four tested applications only had a performance (medical app) or an integrity (airline booking) manipulation, participants were randomly assigned to see a vignette from three of the four applications in order to maintain equal sample sizes across conditions. For each vignette, participants were further randomly allocated to view either a high or low performance or integrity version of the vignette. After reading each vignette, participants rated the perceived risk of using the system, the system's perceived performance, and the system's perceived integrity. Finally, participants provided demographic information including age, gender, and education level.

2.1.4 Results

Across all AI applications mean risk perceptions were greater than 50, indicating that participants perceived some level of risk in each of the depicted scenarios. One-tailed independent samples t-tests of perceived performance conducted between the high and low performance condition of each AI application were statistically significant, indicating that the vignettes as drafted successfully manipulated perceived trustworthiness along the performance dimension. Similarly, one-tailed independent t-tests comparing integrity scores across high and low conditions showed statistically significant differences, indicating successful manipulation of perceived trustworthiness along this dimension.

Supplementary Table 2. Descriptive statistics and t-tests between performance conditions – Pilot Study

AI application	Low performance		High performance		*t*
	M (*SD*)	*n*	*M* (*SD*)	n	(*df*)
Self-driving car	14.00 (18.94)	19	69.76 (16.15)	21	9.97 (35.6)*
Virtual assistant	24.53 (23.37)	19	80.57 (14.19)	21	9.05 (29.09)*
Medical app	12.05 (21.31)	19	66.50 (20.17)	20	8.19 (36.57)*

* $p < .001$

Supplementary Table 3. Descriptive statistics and t-tests between integrity conditions – Pilot Study

AI application	Low integrity		High integrity		*t*
	M (*SD*)	*n*	*M* (*SD*)	*n*	(*df*)
Self-driving car	24.95 (21.95)	20	80.55 (15.94)	20	9.17 (34.68)*
Virtual assistant	16.50 (15.82)	20	73.25 (18.37)	20	10.47 (37.18)*
Airline booking	29.24 (29.74)	21	76.85 (16.42)	20	6.39 (31.46)*

* $p < .001$

3 Supplementary Tables

Supplementary Table 4. Means and Standard Deviations of all Variables by AI Application and Condition: Study 1

	Self-driving car			Virtual assistant			Medical Diagnosis app		
	Total	Low	High	Total	Low	High	Total	Low	High
Trust	3.73	2.95	4.55	4.20	3.38	5.03	3.6	2.66	4.66
	(1.27)	(0.79)	(1.17)	(1.19)	(0.85)	(0.87)	(1.44)	(1.05)	(1.03)
HTP	4.55	4.53	4.57	4.24	4.40	4.09	4.23	4.28	4.18
	(1.36)	(1.26)	(1.46)	(1.49)	(1.41)	(1.56)	(1.60)	(1.54)	(1.68)
MTP	4.84	5.00	4.67	4.89	4.95	4.82	4.64	4.67	4.61
	(1.06)	(0.87)	(1.22)	(1.00)	(0.84)	(1.14)	(1.12)	(1.13)	(1.11)
BI	3.55	2.90	4.23	3.93	2.82	5.04	3.65	2.10	5.21
	(1.75)	(1.44)	(1.88)	(1.75)	(1.53)	(1.15)	(1.99)	(1.52)	(0.85)
N	90	46	44	90	45	45	90	45	45

Note. HTP = Human trust propensity, MTP = Machine trust propensity, BI = Behavioral intention.

Supplementary Table 5. Means and Standard Deviations of All Variables by AI Application and Condition: Study 2

	Self-driving car			Virtual assistant			Airline booking app		
	Total	Low	High	Total	Low	High	Total	Low	High
Trust	3.90	3.03	4.78	3.97	2.83	5.09	4.00	3.18	4.83
	(1.40)	(1.10)	(1.08)	(1.45)	(0.98)	(0.84)	(1.36)	1.32	(0.77)
HTP	4.07	4.23	3.92	4.48	4.65	4.72	4.47	4.49	4.45
	(1.48)	(1.49)	(1.47)	(1.57)	(1.25)	(1.00)	(1.48)	(1.50)	(1.47)
MTP	4.65	4.68	4.62	4.84	4.74	4.94	4.69	4.65	4.72
	(0.98)	(1.08)	(0.89)	(1.22)	(1.27)	(1.16)	(1.13)	(1.25)	(1.00)
BI	4.22	3.42	5.04	4.08	2.92	5.22	4.18	2.93	5.44
	(1.84)	(1.71)	(1.62)	(1.69)	(1.51)	(0.91)	(1.95)	(1.80)	(1.12)
N	91	46	45	89	44	45	90	45	45

Note. HTP = Human trust propensity, MTP = Machine trust propensity, BI = Behavioral intention. For all scales higher scores indicate a higher level of the construct.

Supplementary Table 6. Factor Loadings of Model 1: Study 2

Factor	Item	Standardized loading	SE
Factor 1	TIAS 1	1.00	
	TIAS 2	0.92	0.05
	TIAS 3	0.94	0.05
	TIAS 4	0.91	0.05
	TIAS 5	0.70	0.05
Factor 2	TIAS 6	1.00	
	TIAS 7	0.70	0.05
	TIAS 8	0.86	0.05
	TIAS 9	0.91	0.04
	TIAS 10	0.99	0.04
	TIAS 11	1.061	0.04
	TIAS 12	0.37	0.07

Supplementary Table 7. Summary of Item Analysis Parameters Showing Range of Statistics Across Applications and Studies

TIAS Item	Corr. with total test score	Cronbach's α when item dropped	Average inter-item corr. when item dropped	Corr. of item with BI criterion measure
1	.62 -. 89	.92 - .94	.50 - .60	.53 - .80
2	.46 - .76	.92 - .95	.52 - .62	.31 - .73
3	.68 - .81	.92 - .94	.50 - .59	.55 - .74
4	.71 - .86	.92 - .94	.49 - .59	.63 - .79
5	.57 - .74	.92 - .94	.52 - .61	.43 - .68
6	.83 - .93	.91 - .94	.48 - .58	.77 - .89
7	.54 - .82	.92 - .94	.52 - .60	.59 - .81
8	.63 - .78	.92 - .94	.51 - .60	.53 - .78
9	.75 - .90	.91 - .94	.48 - .59	.74 - .92
10	.76 - .88	.91 - .94	.48 - .59	.71 - .92
11	.84 - .91	.91 - .94	.48 - .57	.81 - .92
12	.33 - .39	.93 - .95	.56 - .65	.29 - .42

Supplementary Table 8. Full Item Analysis Parameters for Study 1: Self-driving Car

TIAS Item	Corr. with total test score	Cronbach's α when item dropped	Average inter-item corr. when item dropped	Corr. of item with BI criterion measure
1	.67	.93	.56	.53
2	.48	.94	.59	.31
3	.70	.93	.56	.65
4	.77	.93	.55	.76
5	.74	.93	.55	.67
6	.88	.92	.53	.77
7	.78	.93	.55	.70
8	.64	.93	.57	.53
9	.86	.93	.54	.74
10	.88	.92	.53	.75
11	.91	.92	.53	.82
12	.35	.94	.61	.42

Supplementary Table 9. Full Item Analysis Parameters for Study 1: Virtual Assistant

TIAS Item	Corr. with total test score	Cronbach's α when item dropped	Average inter-item corr. when item dropped	Corr. of item with BI criterion measure
1	.74	.92	.50	.57
2	.60	.92	.52	.48
3	.68	.92	.50	.55
4	.76	.92	.49	.64
5	.57	.92	.52	.43
6	.83	.91	.48	.81
7	.54	.92	.52	.59
8	.63	.92	.51	.66
9	.86	.91	.48	.79
10	.84	.91	.48	.84
11	.84	.91	.48	.82
12	.33	.93	.56	.29

Supplementary Table 10. Full Item Analysis Parameters for Study 1: Medical Diagnosis App

TIAS Item	Corr. with total test score	Cronbach's α when item dropped	Average inter-item corr. when item dropped	Corr. of item with BI criterion measure
1	.62	.92	.50	.58
2	.46	.92	.52	.37
3	.73	.92	.50	.71
4	.78	.92	.49	.71
5	.73	.92	.52	.68
6	.91	.91	.48	.89
7	.82	.92	.52	.81
8	.78	.92	.51	.77
9	.90	.91	.48	.92
10	.87	.91	.48	.92
11	.91	.91	.48	.89
12	.39	.93	.56	.33

Supplementary Table 8. Full Item Analysis Parameters for Study 2: Self-driving Car

TIAS Item	Corr. with total test score	Cronbach's α when item dropped	Average inter-item corr. when item dropped	Corr. of item with BI criterion measure
1	.67	.93	.56	.53
2	.48	.94	.59	.31
3	.70	.93	.56	.65
4	.77	.93	.55	.76
5	.74	.93	.55	.67
6	.88	.92	.53	.77
7	.78	.93	.55	.70
8	.64	.93	.57	.53
9	.86	.93	.54	.74
10	.88	.92	.53	.75
11	.91	.92	.53	.82
12	.35	.94	.61	.41

Supplementary Table 9. Full Item Analysis Parameters for Study 2: Virtual Assistant

TIAS Item	Corr. with total test score	Cronbach's α when item dropped	Average inter-item corr. when item dropped	Corr. of item with BI criterion measure
1	.84	.93	.57	.73
2	.74	.94	.58	.64
3	.80	.94	.57	.74
4	.711	.94	.59	.63
5	.73	.94	.58	.60
6	.93	.93	.55	.82
7	.57	.94	.60	.62
8	.72	.94	.58	.71
9	.75	.94	.58	.64
10	.87	.93	.56	.82
11	.85	.93	.56	.81
12	.39	.95	.63	.34

Supplementary Table 10. Full Item Analysis Parameters for Study 2: Airline Booking App

TIAS Item	Corr. with total test score	Cronbach's α when item dropped	Average inter-item corr. when item dropped	Corr. of item with BI criterion measure
1	.89	.93	.57	.79
2	.76	.94	.59	.73
3	.81	.94	.59	.74
4	.86	.94	.58	.79
5	.65	.944	.61	.62
6	.83	.94	.58	.79
7	.73	.94	.60	.71
8	.72	.94	.60	.64
9	.75	.94	.59	.64
10	.76	.94	.59	.71
11	.89	.93	.57	.84
12	.35	.95	.65	.36
